# A proposed standard for quantifying 3‐D hindlimb joint poses in living and extinct archosaurs

**DOI:** 10.1111/joa.13635

**Published:** 2022-02-03

**Authors:** Stephen M. Gatesy, Armita R. Manafzadeh, Peter J. Bishop, Morgan L. Turner, Robert E. Kambic, Andrew R. Cuff, John R. Hutchinson

**Affiliations:** ^1^ Department of Ecology, Evolution, and Organismal Biology Brown University Providence Rhode Island USA; ^2^ Structure and Motion Laboratory, Department of Comparative Biomedical Sciences Royal Veterinary College Hatfield UK; ^3^ Museum of Comparative Zoology and Department of Organismic and Evolutionary Biology Harvard University Cambridge Massachusetts USA; ^4^ Geosciences Program, Queensland Museum Brisbane Queensland Australia; ^5^ Department of Computer Science and Engineering University of Minnesota Minneapolis Minnesota USA; ^6^ Department of Biology Hood College Frederick Maryland USA; ^7^ Human Anatomy Resource Centre University of Liverpool Liverpool UK

**Keywords:** comparison, hindlimb, joint, standard, XROMM

## Abstract

The last common ancestor of birds and crocodylians plus all of its descendants (clade Archosauria) dominated terrestrial Mesozoic ecosystems, giving rise to disparate body plans, sizes, and modes of locomotion. As in the fields of vertebrate morphology and paleontology more generally, studies of archosaur skeletal structure have come to depend on tools for acquiring, measuring, and exploring three‐dimensional (3‐D) digital models. Such models, in turn, form the basis for many analyses of musculoskeletal function. A set of shared conventions for describing 3‐D pose (joint or limb configuration) and 3‐D kinematics (change in pose through time) is essential for fostering comparison of posture/movement among such varied species, as well as for maximizing communication among scientists. Following researchers in human biomechanics, we propose a standard methodological approach for measuring the relative position and orientation of the major segments of the archosaur pelvis and hindlimb in 3‐D. We describe the construction of anatomical and joint coordinate systems using the extant guineafowl and alligator as examples. Our new standards are then applied to three extinct taxa sampled from the wider range of morphological, postural, and kinematic variation that has arisen across >250 million years of archosaur evolution. These proposed conventions, and the founding principles upon which they are based, can also serve as starting points for measuring poses between elements within a hindlimb segment, for establishing coordinate systems in the forelimb and axial skeleton, or for applying our archosaurian system more broadly to different vertebrate clades.

## Graphical abstract

We propose a standard approach for measuring the relative position and orientation of the major segments of the archosaur pelvis and hindlimb in 3‐D. A set of shared conventions for describing pose and kinematics is essential for fostering comparison among species and for maximizing communication. We describe the construction of anatomical and joint coordinate systems using the extant guineafowl and alligator as examples and then apply our new standards to three extinct archosaurs.

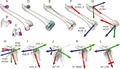

## 1 INTRODUCTION

“Ruling reptiles” (clade Archosauria) dominated terrestrial ecosystems for much of the Mesozoic (Benton & Clark, 1988; Brusatte et al., 2010; Gauthier et al., 1988; Nesbitt, 2011; Nesbitt et al., 2013). From sauropods to hummingbirds, descendants of the most recent common ancestor of crocodylians and birds include species with disparate body plans spanning an immense size range (Benson et al., 2014; Carrano, 2006; Sookias et al., 2012; Turner & Nesbitt, 2013). The significance of evolutionary changes in archosaur hindlimb morphology and function has been the subject of many studies among anatomists, paleontologists, and biomechanists (Bates et al., 2015; Carrano, 2000; Charig, 1972; Gatesy, 1994; Grinham et al., 2019; Hutchinson, 2006; Kubo & Kubo, 2012; Parrish, 1986; Tsai & Holliday, 2015; Walker, 1977). Modifications in limb support (quadruped, biped), foot posture (plantigrade, digitigrade), joint structure, and myology are thought to have important implications for archosaur locomotor performance, behavior, and paleoecology (Bakker, 1971; Bates & Schachner, 2012; Charig, 1972; Gatesy, 1990, 1991; Hutchinson & Gatesy, 2000; Parrish, 1987; Romer, 1923; Rowe, 1986; Sennikov, 1989; Sereno, 1991).

Three‐dimensional (3‐D) digital models have likewise come to dominate morphological analyses of extant and extinct vertebrates in recent years (Davies et al., 2017). Whatever the data source (fossil, osteological, cadaveric, or living specimens), the tools for recreating, comparing, and exploring anatomical structure are becoming more powerful and easier to use (Cunningham et al., 2014; Sutton et al., 2017). The ascendance of 3‐D anatomical studies has been paralleled by 3‐D analyses of musculoskeletal function (e.g. Delp & Loan, 1995; Hutchinson et al., 2008; Seth et al., 2011). High‐resolution reconstructions of 3‐D skeletal kinematics (movement) from biplanar X‐ray video and CT‐based bone models (XROMM: X‐ray reconstruction of moving morphology; Brainerd et al., 2010; Gatesy et al., 2010) have begun to generate rich animations of in vivo behavior from multiple extant taxa. Quantification of 3‐D joint ranges of motion, based either on cadaver tests (Arnold et al., 2014; Cobley et al., 2013; Hutson & Hutson, 2012, 2013; Kambic, Biewener, et al., 2017; Kambic, Roberts, et al., 2017; Manafzadeh, 2020; Manafzadeh & Padian, 2018; Manafzadeh et al., 2021), fossil specimen manipulation (Carpenter & Wilson, 2008; Senter & Robins, 2005), or its virtual counterpart (Demuth et al., 2020; Lai et al., 2018; Mallison, 2010; Nyakatura et al., 2015, 2019; Otero et al., 2017; Pierce et al., 2012; Regnault & Pierce, 2018; Richards et al., 2021; White et al., 2015), is also becoming more common. Augmenting digital skeletal models with digital muscles broadens the spectrum of potential analyses, from investigating 3‐D moment arms (Allen et al., 2021; Bates et al., 2015; Brassey et al., 2017; Hutchinson et al., 2005; O’Neill et al., 2013; Regnault & Pierce, 2018; Sullivan, 2010; Wang et al., 2004; Wiseman et al., 2021) to static or dynamic, optimization‐based simulations of extant and extinct taxa with software such as GaitSym, SIMM, and OpenSim (Bishop, 2019; Bishop, Cuff, et al., 2021; Bishop, Michel, et al., 2021; Bishop et al., 2018a, 2018b; Cox et al., 2019; Heers et al., 2018; Nagano et al., 2005; Rankin et al., 2016; Sellers et al., 2009, 2013, 2017).

**FIGURE 1:**
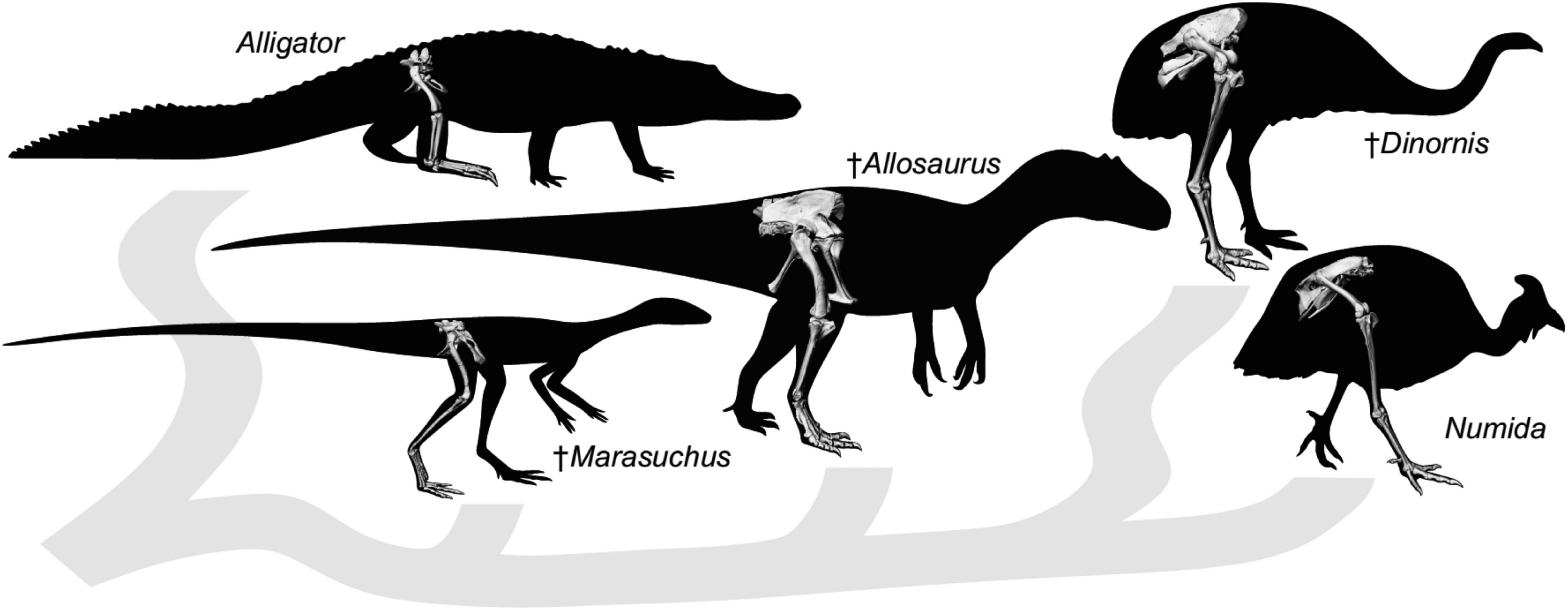
Illustration of various extant and extinct (†) archosaurs, highlighting some of the morphological diversity observed in the pelvis and hindlimb within the group. General phylogenetic relationships among the taxa are also shown. Alligator (*Alligator*) is an extant representative of Pseudosuchia, Guineafowl (*Numida*) is an extant representative of Neognathae (Aves), *Marasuchus*/*Lagosuchus* is an extinct dinosauriform, *Allosaurus* an extinct theropod, and *Dinornis* is an extinct representative of Palaeognathae (Aves). Not to scale

As 3‐D analyses become ubiquitous, the benefits of standards for describing 3‐D pose (joint or limb configuration) and 3‐D kinematics (change in pose through time) become obvious. Establishing consistent procedures for quantifying articular relationships fosters comparison, particularly among disparate species. Moreover, if different workers describe joint pose/kinematics using different measurement methods, the likelihood of meaningful quantitative comparison among 3‐D datasets becomes remote, analogous to using non‐homologous morphometric landmarks. Researchers in human biomechanics recognized this problem and successfully established conventions for measuring 3‐D joint translations and rotations from the relative position and orientation of adjacent bones. In a series of papers, the Standardization and Terminology Committee of the International Society of Biomechanics laid out recommendations for measuring major joints of the limbs and spine (Wu et al., 2002, 2005) based on the knee joint coordinate system (JCS) proposed by Grood and Suntay (1983). Herein, our more ambitious aim is to create a comparable system of standards that can encompass the much wider range of anatomical, postural, and kinematic variation that has arisen across >250 million years of archosaur evolution (Figure 1). With the full acknowledgment that this first effort will be incomplete and leave some issues unresolved, we offer our recommendations as a common starting point.

The goal of this paper is to propose a standard methodological approach for measuring the relative position and orientation of the major segments of the archosaur pelvis and hindlimb in 3‐D. We begin by describing the founding principles that guided the creation of our standard. We then review the basic elements of JCSs and introduce how we implement our approach. Given this background, we present a description of our standard for the pelvis, femur, crus, and foot of extant archosaurs, and demonstrate its applicability to a sample of extinct taxa. Finally, we discuss the known shortcomings and benefits of our proposed standard and offer potential next steps for moving forward.

## 2 METHODS

### 2.1 Founding principles

We established our proposed standard based on five founding principles. First and foremost, we strove to create a system with sufficient flexibility to *accommodate a broad disparity* in hindlimb morphologies, sources of data, and applications. We want the standard to be a useful starting point for researchers quantifying 3‐D kinematics in extant species, as well as those working to reanimate movement in their extinct relatives.

Second, we sought a protocol that was *established on static morphology*, such that poses could be calculable from a single configuration (as for fossils in situ, mounted skeletons, CT‐scanned cadavers/subjects, or single frames of X‐ray video) without requiring movement data. Methods that quantify bone relationships based on in vivo motion or movement during cadaveric manipulation (e.g., instantaneous axes or helical axes of rotation; Fuller et al., 1997; Horsman et al., 2007; Rubenson et al., 2007) were precluded.

Third, we explicitly chose to *focus on the bony skeleton* so that the standard could encompass both extant and extinct archosaurs, the latter being typically known only by their ossified elements. Although models that include articular cartilage (e.g. Tsai, Turner, et al., 2020) can be easily handled with the same protocol, this alternative is not explored here.

Fourth, we took a *joint‐inspired, segment‐based approach* to quantify hindlimb poses. Each segment (pelvis, femur, crus, foot) is treated as a unit that is represented, in simplified form, as anatomically derived long‐ and transverse axes. This T‐shaped pair of axes forms the foundation for creating each segment’s proximal and distal coordinate systems, which are then combined with those of adjacent segments to measure rotations and translations.

Finally, we chose to quantify joint rotations using *Euler/Tait–Bryan angles*. Joint poses are described with three rotational degrees of freedom: flexion–extension (FE), abduction–adduction (ABAD), and long‐axis rotation (LAR) about the *Z*‐, *Y*‐, and *X*‐axes, respectively. In contrast to other 3‐D rotation representations (e.g., matrices, axis–angle, helical axis, quaternions), Euler/Tait–Bryan angles are the most intuitive representation of joint pose in an anatomical context, making them the recommended format of the International Society of Biomechanics for human kinematic data (Wu et al., 2002, 2005). We likewise believe that, despite some drawbacks (discussed below), Euler/Tait–Bryan angles are most accessible to biologists and paleontologists not specializing in 3‐D kinematic analysis.

### 2.2 Overview of joint and anatomical coordinate systems

**FIGURE 2:**
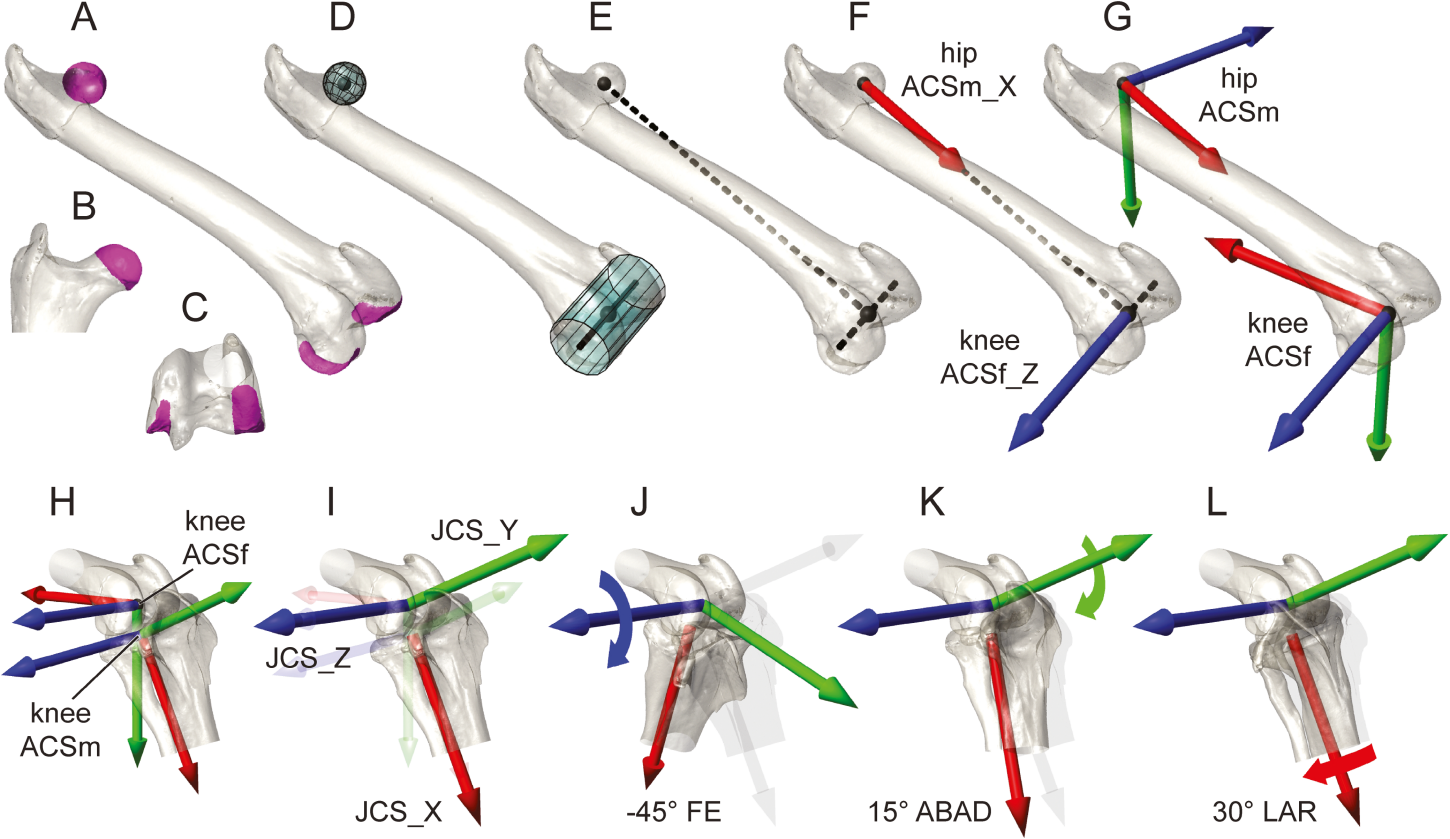
Steps in anatomical coordinate system (ACS) creation (a–g), joint coordinate system (JCS) creation (h–i), and example rotations (j–l), using a guineafowl right femur and right knee as examples. Selected articular surface patches (magenta, a–c) are fit with spherical (head) and cylindrical (condyles) geometric primitives (aqua, d). An intercentroid long‐axis vector, along with the cylinder axis, form a T‐shaped pair of principal vectors (dashed, e) from which cardinal axes (f) are derived. Proximal (hipACSm) and distal (kneeACSf) ACS origins are at the sphere and cylinder centroids, respectively. ACS axes (g) are derived from the cardinal axes by vector math. Fixed (kneeACSf) and mobile (kneeACSm) axes are shown for the distal femur and proximal crus in an extended knee pose (h). Axes of the knee JCS are shown slightly larger relative to their underlying, transparent ACS axes (i). The hierarchical nature of JCS rotations is shown by changes in bones and axes caused by rotation about flexion–extension (FE) (j), abduction–adduction (ABAD) (k), and long‐axis rotation (LAR) (l) axes

Grood and Suntay (1983) presented a specific coordinate system for measuring 3‐D motion of the human knee. Their JCS is easily generalized, has been widely adopted for humans and other taxa (e.g., Baier & Gatesy, 2013; Baier et al., 2013; Bhullar et al., 2019; Bishop, Cuff, et al., 2021; Bishop, Michel, et al., 2021; Gidmark et al., 2012; Heers et al., 2016; Kambic, Biewener, et al., 2017; Kambic, Roberts, et al., 2017; Kambic et al., 2014, 2015; Menegaz et al., 2015; Miranda et al., 2013; Provini & Abourachid, 2018; van Meer et al., 2019; Wiseman et al., 2021), and serves as the basis for our proposed standard (Figure 2). A JCS consists of a set of explicitly defined axes for measuring the translations and rotations between two bodies. Limbs are typically analyzed as a kinematic chain, using a JCS to measure the pose of a distal “child” segment relative to its proximal “parent” segment (e.g., the crus relative to the femur). JCS axes are themselves derived from a pair of 3‐D Cartesian coordinate systems (one per body), each composed of an origin and three orthogonal axes (Figure 2g). Throughout this paper we will use a consistent coloring scheme in which *X*‐axes are red, *Y*‐axes are green, and *Z*‐axes are blue (easily remembered as *XYZ* = RGB). Because biologists and biomechanists position and orient such sets of axes on anatomical structures, they are referred to as anatomical coordinate systems (ACSs). Thus, in order to create JCSs to measure archosaur hindlimbs, we must first establish pairs of ACSs, one on either side of each major joint (Figure 2h). For each JCS, we designate the proximal ACS as “fixed” (ACSf) and the distal ACS as “mobile” (ACSm) to reflect their parent–child relationship even as both move in space locked to their respective segments.

Anatomical coordinate systems can be created for skeletal elements using a variety of morphological criteria. One method treats a 3‐D bone model as solid in order to calculate its virtual center of mass and inertial axes (Coburn et al., 2007; Crisco & McGovern, 1998). Although this procedure is objective and highly reproducible (the same model should yield identical results every time), specific anatomical features are not incorporated. Alternatively, ACSs can be made using homologous bony features in the same way that external motion capture markers are applied over palpable landmarks (Wu et al., 2002). Yet another technique employs specific surface patches composed of a subset of the model’s polygons and vertices to characterize joint geometry (Miranda et al., 2013; Renault et al., 2018). Many methods used for archosaur limbs apply a combination of these approaches (Baier & Gatesy, 2013; Baier et al., 2013; Bishop, Cuff, et al., 2021; Bishop, Michel, et al., 2021; Hutchinson et al., 2005, 2015; Kambic et al., 2014; Otero et al., 2017; Suzuki et al., 2021). In every case, care must be taken to ensure that the resulting coordinate systems have been implemented correctly according to what is desired (see Bates et al., 2015).

Herein we exclusively employ the fitting of geometric primitives to surface patches selected from the proximal and distal articular surfaces (following Eckhoff et al., 2001). We choose a primitive type (sphere, cylinder, or plane) based on overall similarity and inferred motion at the joint. For example, we fit the biconvex femoral head with a sphere, whereas we fit the roller‐like distal femoral condyles with a cylinder (Figure 2a–d). For all but the sacral vertebrae, surface patches are portions of the bone model known (or presumed) to support articular cartilage. We manually select polygons to include in surface patches, making this a subjective step in the process. Geometric primitives can be fit by eye (Costa et al., 2014; Lai et al., 2018; Molnar et al., 2021; Pierce et al., 2012), but we prefer automated solutions to optimize their scale, position, and orientation. Tools for automated fitting of geometric primitives to surface patches are available in several commercial software packages (e.g., Geomagic, 3‐Matic, Rhino) or online (e.g., MATLAB scripts made available by Bishop, Cuff, et al., 2021; Modenese & Renault, 2021; Renault et al., 2018).

Our general approach for transforming surface patches into ACSs can be described using a CT scan‐derived polygonal model of a right guineafowl femur as an example (Figure 2a–g). Two ACSs are needed: one for measuring femoral pose relative to the pelvis at the hip (hipACSm), and a second for measuring pose of the crus relative to the femur at the knee (kneeACSf). The origins of the hipACSm and kneeACSf are positioned inside the femur at the centroids of the fit head sphere and fit condyle cylinder, respectively (Figure 2d). The axes of both ACSs are then constructed by basic vector algebra. First, we create a femoral long‐axis vector between the sphere and cylinder centroids by subtracting their 3‐D coordinates. When this femoral long‐axis vector is combined with the fit femoral condyle cylinder axis (which need not be perpendicular), the pair serve as T‐shaped principal vectors (dashed lines in Figure 2e) from which two principal axes (Figure 2f) and all other ACS axes are derived.

Once normalized to unit length, the long‐axis vector becomes the *X*‐axis of the mobile hip ACS (hipACSm_X; Figure 2f). The femoral cylinder axis, in unitized vector form, becomes the *Z*‐axis of the fixed knee ACS (kneeACSf_Z; Figure 2f). A sequence of principal axis cross‐product calculations yields two new axes orthogonal to the plane of the “T” (green *Y*‐axes; Figure 2g), which are then crossed with their respective principal axes to create both complete ACS triads (Figure 2g). Other limb segments follow the same pattern as the femur (Method S1); only the derivation of the pelvicACSm differs slightly (see below).

After ACSs are constructed for all segments, JCSs are defined using fixed and mobile pairs at each joint. For example, the knee JCS measures deviation of the kneeACSm on the proximal crus relative to the kneeACSf on the distal femur (Figure 2h). When these ACSs are perfectly registered (origins and axes in complete alignment), there is no translation or rotation of the JCS. We refer to this as the joint’s reference pose and say that it is “zeroed out”; all six degrees of freedom have value 0. If all joints are in our proposed reference pose, the hindlimb is fully collapsed into an unrealistic configuration in which bone models interpenetrate. Natural poses entail JCS translation and rotation away from the reference pose as each ACSm deviates from its respective ACSf.

Joint coordinate system rotations are calculated about two axes that remain static relative to their segments and a third, mobile axis (Figure 2i). FE takes place about the *Z*‐axis of the JCS (JCS_Z), which is identical to the *Z*‐axis of the ACSf (ACSf_Z). At the knee (Figure 2j), FE measures rotation of the ACSm (on the crus) about the ACSf_Z (on the femur). Recall that kneeACSf_Z equates with the axis of the fit cylinder, which is a logical choice given that the crus likely follows the arc of the femoral condyles during FE. LAR takes place about the *X*‐axis of the JCS (JCS_X), which is the same as the *X*‐axis of the ACSm (ACSm_X). At the knee (Figure 2l), LAR measures rotation of the ACSm (on the crus) about its own ACSm_X. KneeACSm_X equates with the crural long‐axis vector, and thus serves as the ideal JCS axis for measuring long‐axis “spin” of the crus.

Abduction–adduction is not measured about any of the body‐embedded ACS axes. Rather, a new, dynamic *Y*‐axis (JCS_Y) is created that remains orthogonal to both the *Z*‐ and *X*‐axes just described. At the knee (Figure 2k), ABAD measures rotation of the ACSm (on the crus) about this “floating” *Y*‐axis. Perhaps surprisingly, JCS_Z and JCS_X need not remain perpendicular (Grood & Suntay, 1983). During normal functioning of the JCS, any ABAD away from the reference of 0° entails reducing the angle between JCS_Z and JCS_X to greater or less than 90°.

Our hip, knee, and ankle JCSs describe joint pose and motion using conventional zoological (and clinical) terms for tetrapods with relatively erect hindlimb posture: FE, ABAD, and LAR measure rotations about JCS_Z, JCS_Y, and JCS_X, respectively (Figure 2j–l). To describe pelvic position and orientation in global space, we adapt the JCS approach by creating a pseudo‐joint between the pelvis and a non‐anatomical fixed ACS (pelvicACSf) anchored on the ground external to the animal. The corresponding mobile ACS on the pelvis (pelvicACSm) is placed to allow pelvic rotations to be measured as yaw (about JCS_Z), pitch (about JCS_Y), and roll (about JCS_X).

We do not have sufficient space to fully explain the geometry of JCS axes and Euler/Tait–Bryan angles here, but a brief analogy may help clarify their relationship. The axes of the JCS are arranged hierarchically into a rigid rotation order (in our case, *ZYX*) akin to a set of nested gimbals. At the bottom of the hierarchy, LAR about JCS_X affects only the distal segment (Figure 2l). ABAD about JCS_Y affects both JCS_X and the distal segment (Figure 2k). At the top of the hierarchy, FE about JCS_Z affects JCS_Y, JCS_X, and the distal segment (Figure 2j). As a result of this relationship, issues may arise when ABAD or pelvic pitch approach extreme values near ±90° (see Section 4).

JCS translations (the Euclidean distance between mobile and fixed ACS origins) have been expressed in various ways across existing kinematic studies. Here we deviate from Grood and Suntay (1983) in not measuring the *X*, *Y*, and *Z* components of translation along the axes of the JCS itself, since these may not always be mutually orthogonal. Instead, we follow the relatively straightforward approach of previous XROMM and multibody simulation studies, which measure the components of the translation vector along the axes of the JCS’s fixed ACS (e.g., kneeACSf). Pelvic translations are simply displacements of pelvicACSm from pelvicACSf along the axes of the ground‐based ACSf. Future studies may also benefit from creating specialized translation parameterizations, as Manafzadeh and Gatesy (2021) recently developed for hinge‐like joints, to best address the research question at hand.

### 2.3 Standards presentation

We present our archosaur ACS, JCS, and reference pose standards with figures of a Helmeted Guineafowl (*Numida meleagris*) and an American alligator (*Alligator mississippiensis*). These representatives of the two archosaur crown clades are referred to as “guineafowl” and “alligator” for simplicity. We likewise minimize excessive repetition by omitting vector normalization; all vectors should be unit length when constructing ACS axes. Guineafowl and alligator figures were constructed in Adobe Illustrator CC 2020 and Photoshop CC 2020 using renderings from Autodesk Maya 2020, within which coordinate system models were created and placed. Patch selection and primitive fitting were done with Geomagic Studio 2013 and Geomagic Wrap 2017 (3D Systems) using .obj format polygonal models created in Amira 6.0 (Thermo Fisher Scientific). Scans of guineafowl and alligator elements were made with a Nikon Xtek microCT (Nikon Metrology) at 115–120 kV, 125–130 μA, 0.063–0.090 mm slice thickness, and 2000 × 2000 resolution.

## 3 RESULTS

### 3.1 ACS standards

**FIGURE 3:**
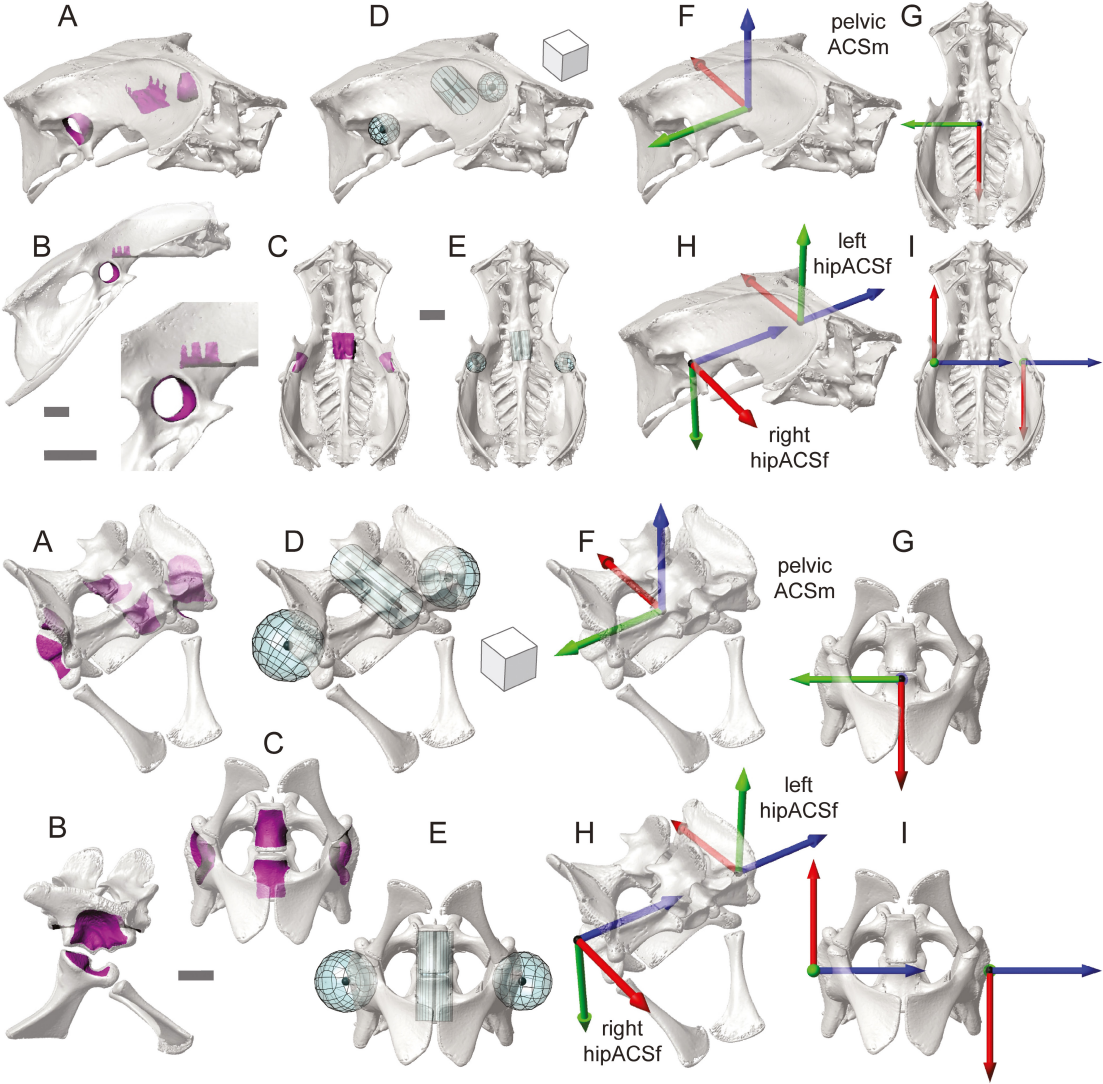
Anatomical coordinate system (ACS) standards for the pelvis, as demonstrated by guineafowl (a–i, top) and alligator (a–i, bottom). Selected surface patches (magenta) for the acetabula and sacral centra in oblique (a), right lateral (b), and ventral (c) views. Fit acetabular spheres and sacral cylinders (aqua) in oblique (d) and ventral (e) views. Resulting pelvicACSm (f, g) and hipACSf (h, i) coordinate systems in oblique (f, h) and ventral (g, i) views. Sphere centroids are shown in black. Scale bars and cube sides equal 1 cm

**FIGURE 4:**
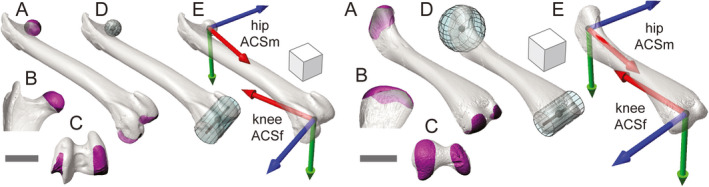
Anatomical coordinate system (ACS) standards for the right femur, as demonstrated by guineafowl (a–e, left) and alligator (a–e, right). Selected surface patches (magenta) for the femoral head and condyles in oblique (a), cranial (b), and distal (c) views. Fit head spheres and condylar cylinders (aqua) in oblique (d) views. Resulting hipACSm and kneeACSf coordinate systems in oblique (e) views. Sphere and cylinder centroids are shown in black. Scale bars and cube sides equal 1 cm

**FIGURE 5:**
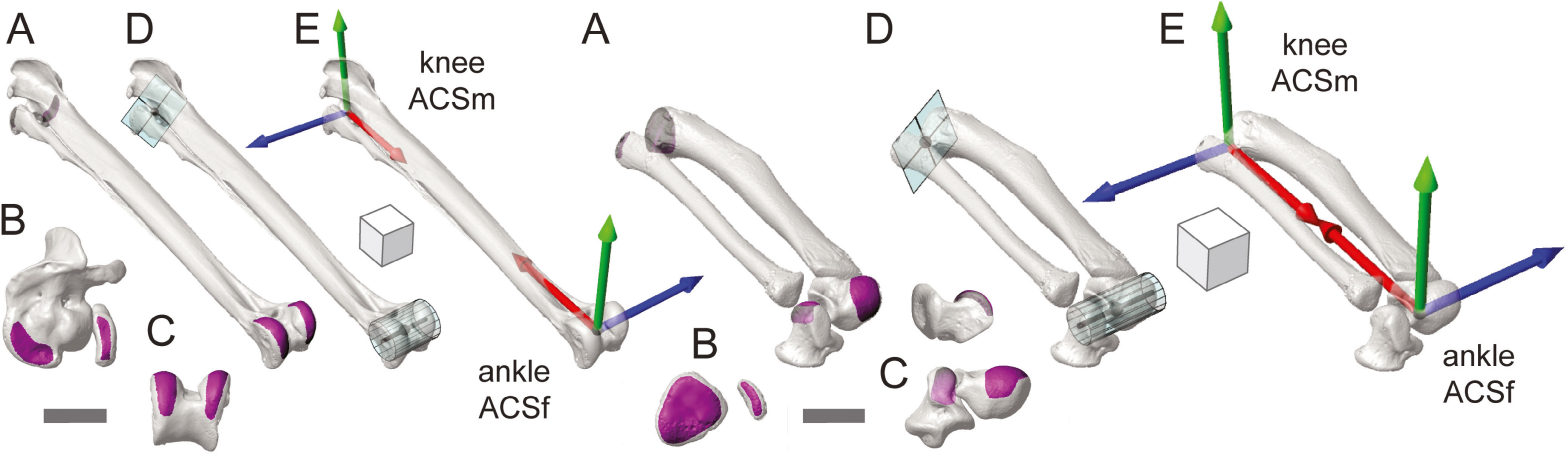
Anatomical coordinate system (ACS) standards for the right crus, as demonstrated by guineafowl (a–e, left) and alligator (a–e, right). Selected surface patches (magenta) for the tibial and fibular heads, tibiotarsal condyles, and astragalo‐calcaneal rollers in oblique (a), proximal (b), and distal (c) views. An additional lateral view of the alligator calcaneum is included (c, above at right). Fit proximal planes and distal cylinders (aqua) in oblique (d) views. Resulting kneeACSm and ankleACSf coordinate systems in oblique (e) views. Plane and cylinder centroids are shown in black. Scale bars and cube sides equal 1 cm

**FIGURE 6:**
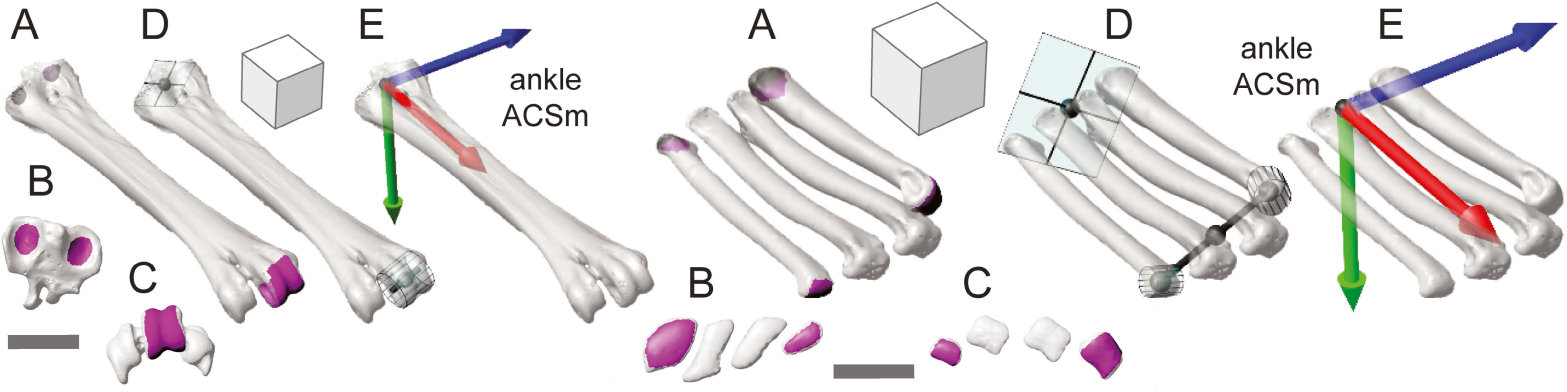
Anatomical coordinate system (ACS) standards for the right foot, as demonstrated by guineafowl (a–e, left) and alligator (a–e, right). Selected surface patches (magenta) for the proximal and distal articular surfaces in oblique (a), proximal (b), and distal (c) views. Fit proximal planes and distal cylinders (aqua) in oblique (d) views. The third metatarsal condyle is used in guineafowl, whereas a mid‐condyle point halfway between the outer metatarsals serves as a dynamic distal centroid in alligator (d). Resulting ankleACSm coordinate systems in oblique (e) views. Plane, cylinder, and mid‐condyle centroids are shown in black. Scale bars and cube sides equal 1 cm

Instructions for creating ACSs are presented in turn, proceeding from proximal to distal for guineafowl and alligator. Within the pelvic (Figure 3), femoral (Figure 4), crural (Figure 5), and foot/pedal (Figure 6) segments, we first describe polygonal patch selection and primitive fitting, followed by the steps for establishing the ACS origins and axes. Right limb elements are figured, with guineafowl and alligator presented in parallel using the same figure labels (i.e., Figure 3a designates pelvic patch selections in oblique view for both species). ACS calculations for the left side are described separately because our ACSs are asymmetric.

#### 3.1.1 Pelvic

Starting with select polygonal patches (Figure 3a–c), three primitives (Figure 3d,e) are created to establish three ACSs (Figure 3f–i) on the pelvis: a pelvicACSm, right hipACSf, and left hipACSf. We first select polygonal faces comprising the wall of each acetabulum and fit them each with a sphere; the articular surface of the antitrochanter in the guineafowl is not included. Polygonal faces comprising the sacral centra are then fit with a cylinder (axis positive cranially). The goal of this cylinder is to serve as a longitudinal sacral axis in the region of the acetabulum, so we do not consider specific vertebral homology to be essential. Only two sacrals are present in alligator, but for guineafowl we use the fourth through seventh to provide adequate length.

To establish pelvicACSm (Figure 3f,g), we create an origin midway between the acetabular sphere centroids. PelvicACSm_Y is created by subtracting the left acetabular centroid from the right acetabular centroid (positive to the right). The sacral cylinder axis vector and interacetabular vector (pelvicACSm_Y) form the T‐shaped principal vectors from which the *Z*‐ and *X*‐axes are established. PelvicACSm_Y is crossed by the sacral cylinder axis vector, yielding pelvicACSm_Z (positive dorsally). Finally, crossing the *Y*‐axis by the *Z*‐axis yields pelvicACSm_X (positive caudally).

The counterpart to pelvicACSm, pelvicACSf, is a non‐anatomical, externally‐placed coordinate system from which motion of the pelvis is referenced. For terrestrial locomotion, pelvicACSf has its origin on the surface of the ground and a vertical pelvicACSf_Z. PelvicACSf_X and pelvicACSf_Y are both horizontal and perpendicular, but their exact orientations in world space are user‐defined.

We also create ACSs at each acetabular centroid to measure hip movement (Figure 3h,i). For both right and left acetabular ACSs, hipACSf_Z is calculated by subtracting the right acetabular centroid from the left acetabular centroid (both sides positive to the left). On the right side, we then cross this *Z*‐axis by the sacral cylinder axis vector to create hipACSf_Y (positive ventrally). On the left side, we reverse the crossing order to make the *Y*‐axis positive dorsally. For both sides, the *Y*‐axis is then crossed by the *Z*‐axis, yielding hipACSf_X (right positive cranially, left positive caudally).

#### 3.1.2 Femoral

ACSs for the right femur in guineafowl were introduced previously (Figure 2); we present the complete standard here. Based on select polygonal faces (Figure 4a–c) and fit primitives (Figure 4d), we make two ACSs for each femur (Figure 4e): a hipACSm proximally and a kneeACSf distally. After isolating polygonal faces comprising the femoral head, we fit them with a sphere. The centroid of this sphere forms the hipACSm origin. Distally, we select polygons comprising the femoral condyles (not including the tibiofibular condyle in guineafowl) and fit them with a cylinder (right axis positive laterally, left axis positive medially). The centroid of this cylinder forms the kneeACSf origin.

On the right side, we subtract the origin of hipACSm from that of kneeACSf to calculate hipACSm_X (positive distally). On the left side, the subtraction order is reversed (hipACSm_X positive proximally). Each femoral cylinder’s axis designates a kneeACSf_Z (right positive laterally, left positive medially). We calculate the proximal and distal *Y*‐axes by crossing hipACSm_X by kneeACSf_Z on both sides (right positive caudally, left positive cranially). Crossing each femur’s kneeACSf_Y by its kneeACSf_Z then yields its kneeACSf_X (right positive proximally, left positive distally). Finally, we create hipACSm_Z for each side by crossing hipACSm_X by hipACSm_Y (right positive medially, left positive laterally).

#### 3.1.3 Crural

Our “crural” segment (tibia, fibula, and proximal tarsals) exhibits greater variation than the femoral segment among archosaurs. The calcaneum, in particular, presents complications for comparing mesotarsal and crurotarsal ankle morphologies (Schaeffer, 1941; Brinkman, 1980; Cruickshank, 1979; Cruickshank & Benton, 1985; Demuth et al., 2020; Parrish, 1987, 1993; Sereno & Arcucci, 1990; see Section 4). Despite significant variation in calcaneal mobility, our approach to crural ACS creation only differs between guineafowl and alligator in minor detail. Based on selected polygonal patches (Figure 5a–c) and fit primitives (Figure 5d), we make two ACSs for each crus (Figure 5e): a kneeACSm proximally and an ankleACSf distally. After isolating polygonal faces comprising the proximal articular surfaces of the fibula and either tibiotarsus (guineafowl) or tibia (alligator), we fit them with a plane. Note that for the avian tibiotarsus, only the articular surface of the medial facies is selected; the interarticular eminence and cnemial crest are excluded as they do not engage the femoral condyles. The centroid of this plane forms the kneeACSm origin.

Distally, we fit a cylinder to both mesotarsal and crurotarsal morphologies. In the mesotarsal guineafowl, polygons comprising the roller‐like tibiotarsal condyles (Figure 5c,d; left) are selected (focusing purely on the surfaces expected to contact the tarsometatarsal cotylar surfaces) and fit with a cylinder. Perhaps counterintuitively, the proximal tarsals of the crurotarsal alligator also approximate a cylindrical pair. The convex, distal surface of the astragalus is selected along with the roller‐like *proximal* surface of the calcaneum that articulates with the fibula (Figure 5c,d; right), as fibular–calcaneal movement in crocodylians is demonstrably (Brinkman, 1980) greater than that between calcaneum and distal tarsals. For the alligator, our cylinder fitting thus aims to find a transverse astragalo‐calcaneal axis akin to the pin joining adjacent “knuckles” of a door hinge. Although this axis is not completely stable, the benefits of including the calcaneum in forming a transverse crural vector outweigh the costs in our simplified, segment‐based system. The centroid of the fit cylinder (right axis positive medially, left axis positive laterally) forms the ankleACSf origin.

As with the femoral sphere and cylinder, the crural plane and cylinder also give rise to a pair of T‐shaped principal vectors. On the right side, we subtract the origin of kneeACSm from that of ankleACSf to calculate the crural long axis vector and kneeACSm_X (positive distally). On the left side, the subtraction order is reversed (kneeACSm_X positive proximally). Each cylinder’s axis vector designates an ankleACSf_Z (right positive medially, left positive laterally). We calculate the proximal and distal *Y*‐axes by crossing kneeACSm_X by ankleACSf_Z on both sides (right positive cranially, left positive caudally). Crossing each crus’ ankleACSf_Y by its ankleACSf_Z then yields its ankleACSf_X (right positive proximally, left positive distally). Finally, we create kneeACSm_Z by crossing kneeACSm_X by kneeACSm_Y (right positive laterally, left positive medially).

#### 3.1.4 Pedal

Variation in foot morphology raises the greatest challenge to creating a common pedal coordinate system. Calculation of an ACS for the guineafowl tarsometatarsus is relatively straightforward, following the same approach employed previously. A plane can be fit to selected polygons on the proximal articular surfaces of the fused tarsals. To create the T‐shaped principal vectors, a distal centroid and transverse axis vector can be developed based on the condyle of metatarsal III. However, the alligator’s unfused tarsals, mobile metatarsals, and four weight‐bearing toes are not amenable to identical treatment; there is no single dominant digit. Given the goal of a segment‐level standard, several simplifications and accommodations are required.

First, unfused distal tarsals are excluded from ACS creation. Given current uncertainty about the contribution of distal tarsals to ankle movement in extant crocodylians and their variable preservation, ossification, and fusion in fossils (Cruickshank, 1979; Holtz, 1995; Müller & Alberch, 1990; Ossa‐Fuentes et al., 2022; Padian, 2017; Sullivan, 2010), we use only the metatarsals. Second, we drop our preference for strict homology. The concession we make is to choose the outermost (medial‐most and lateral‐most) pair of main metatarsals for polygonal patch selection and primitive fitting, regardless of number. Thus we use I and IV for alligator and III for guineafowl. Third, whereas the guineafowl ACS maintains a static relationship to the tarsometatarsus, our alligator coordinate system is dynamic. Unfused, loosely bound metatarsals are able to spread, skew, and long‐axis rotate within the foot (Brinkman, 1980; Turner & Gatesy, 2021). Although the foot can be treated statically by selecting a single metatarsal (e.g., metatarsal III; Wiseman et al., 2021) our dynamic approach can better reflect overall foot pose by using the position of both outermost condyles to define a pedal transverse axis. The latter point is a particular benefit, as metatarsal condylar axes rotate up to 60° inside the foot and rarely align with the pedal transverse axis (Turner & Gatesy, 2021). Under this dynamic approach, we recalculate the T‐shaped pair of principal vectors on a per‐pose basis, thereby incorporating at least some aspects of intermetatarsal mobility into the measurement of ankle motion.

We make a single ACS, ankleACSm, for each foot. After isolating polygonal faces (Figure 6a,b) comprising the proximal articular surface of the tarsometatarsus (excluding the intercotylar eminence in guineafowl) or proximal articular surfaces of metatarsals I and IV (alligator), we fit them with a plane (Figure 6d). The centroid of this plane forms the ankleACSm origin. We create the other elements required for our principal vectors in two ways, depending on foot morphology. In guineafowl and other taxa with fused or tightly bound metatarsals, we fit a cylinder to the articular polygonal faces of tarsometatarsal condyle III (Figure 6a,c,d; left). In alligator, articular polygonal faces comprising metatarsal condyles I and IV are selected and fit with separate cylinders (Figure 6a,c,d; right). The mid‐point between these two cylinder centroids is used in calculating the pedal long‐axis vector (with the proximal plane centroid), whereas a vector from one centroid to the other serves as the pedal transverse axis (Figure 6d; right). On the right foot, the medial cylinder centroid is subtracted from the lateral cylinder centroid (transverse axis vector positive laterally); on the left foot the subtraction order is reversed (transverse axis vector positive medially).

Pedal ACS axes (Figure 6e) are created as for the other segments. On the right side, we subtract the origin of ankleACSm from the distal condylar centroid or mid‐point to calculate ankleACSm_X (positive distally). On the left side, the subtraction order is reversed (positive proximally). Crossing ankleACSm_X by the pedal transverse axis vector yields ankleACSm_Y (right positive caudally, left positive cranially). Finally, we create ankleACSm_Z by crossing ankleACSm_X by ankleACSm_Y (right positive medially, left positive laterally).

Under our dynamic approach, the requirement to recalculate the ankleACSm in alligator (and other archosaurs with “free” metatarsals) each time the foot moves is not particularly arduous for quantifying several poses. However, the burden increases for kinematic analyses involving hundreds or thousands of pedal configurations. In particular, confidently tracking the LAR of the medial‐ and lateral‐most metatarsals is a challenge that can be avoided with little loss of information, as only the condylar centroids are used for our ACS. Although condylar cylinders are useful for creating coordinate systems for metatarsophalangeal joints (not presented here), a comparable pedal transverse axis can be constructed by any pair of consistently trackable points on the outermost metatarsals (implanted radiopaque markers, collateral ligament fossae, etc.).

### 3.2 JCS standards and reference poses

As described in the Overview section of Methods, each JCS is created from a pair of ACSs, one fixed (ACSf) and the other mobile (ACSm). JCS_Z is equivalent to ACSf_Z; JCS_X is equivalent to ACSm_X; and JCS_Y “floats” to remain perpendicular to both. In this section, we describe the rotations at each joint and their signs, following a previously published scheme for guineafowl (Kambic et al., 2014). Because our hip, knee, and ankle ACSs are created asymmetrically, subsequent JCS rotations have the same sign for equivalent anatomical motion of both right and left hindlimbs.

#### 3.2.1 Pelvic

We create the pelvic JCS from the pelvicACSf_Z on the ground, the pelvicACSm_X along the sacral vertebrae, and a floating *Y*‐axis that stays perpendicular to both. Rotation about pelvicJCS_Z denotes yaw; in dorsal view, turning counter‐clockwise (left) is positive. Rotation about pelvicJCS_X denotes roll; raising the right acetabulum relative to the left is positive. Rotation about the floating *Y*‐axis (pelvicJCS_Y) represents pitch; raising the cranial end is positive. Translations measure displacements of the pelvicACSm origin from the pelvicACSf origin along the pelvicACSf axes.

#### 3.2.2 Hip

We create each hip JCS from the hipACSf_Z at the acetabulum, the hipACSm_X at the femoral head, and a floating *Y*‐axis that stays perpendicular to both. Rotation about the hipJCS_Z denotes FE; extension is positive. Rotation of the femur about hipJCS_X denotes LAR; external LAR is positive. Rotation about the floating *Y*‐axis (hipJCS_Y) denotes ABAD; abduction is positive. Hip translations measure displacements of the hipJCSm origin from the hipJCSf origin along the axes of hipACSf.

#### 3.2.3 Knee

We create each knee JCS from the kneeACSf_Z at the femoral condyles, the kneeACSm_X at the proximal crus, and a floating *Y*‐axis that stays perpendicular to both. Rotation about kneeJCS_Z denotes FE; extension is positive. Rotation of the crural segment about kneeJCS_X denotes LAR; external LAR is positive. Rotation about the floating *Y*‐axis (kneeJCS_Y) denotes ABAD; adduction is positive. Knee translations measure displacements of the kneeJCSm origin from the kneeJCSf origin along the axes of kneeACSf.

#### 3.2.4 Ankle

We create each ankle JCS from the ankleACSf_Z at the distal crus, the ankleACSm_X at the proximal foot, and a floating *Y*‐axis that stays perpendicular to both. Rotation about ankleJCS_Z denotes FE; extension is positive. Rotation of the foot about ankleJCS_X denotes LAR; external LAR is positive. Rotation about the floating *Y*‐axis (ankleJCS_Y) denotes ABAD; abduction is positive. Ankle translations measure displacements of the ankleJCSm origin from the ankleJCSf along the axes of ankleACSf.

#### 3.2.5 Reference poses and JCS representation

**FIGURE 7:**
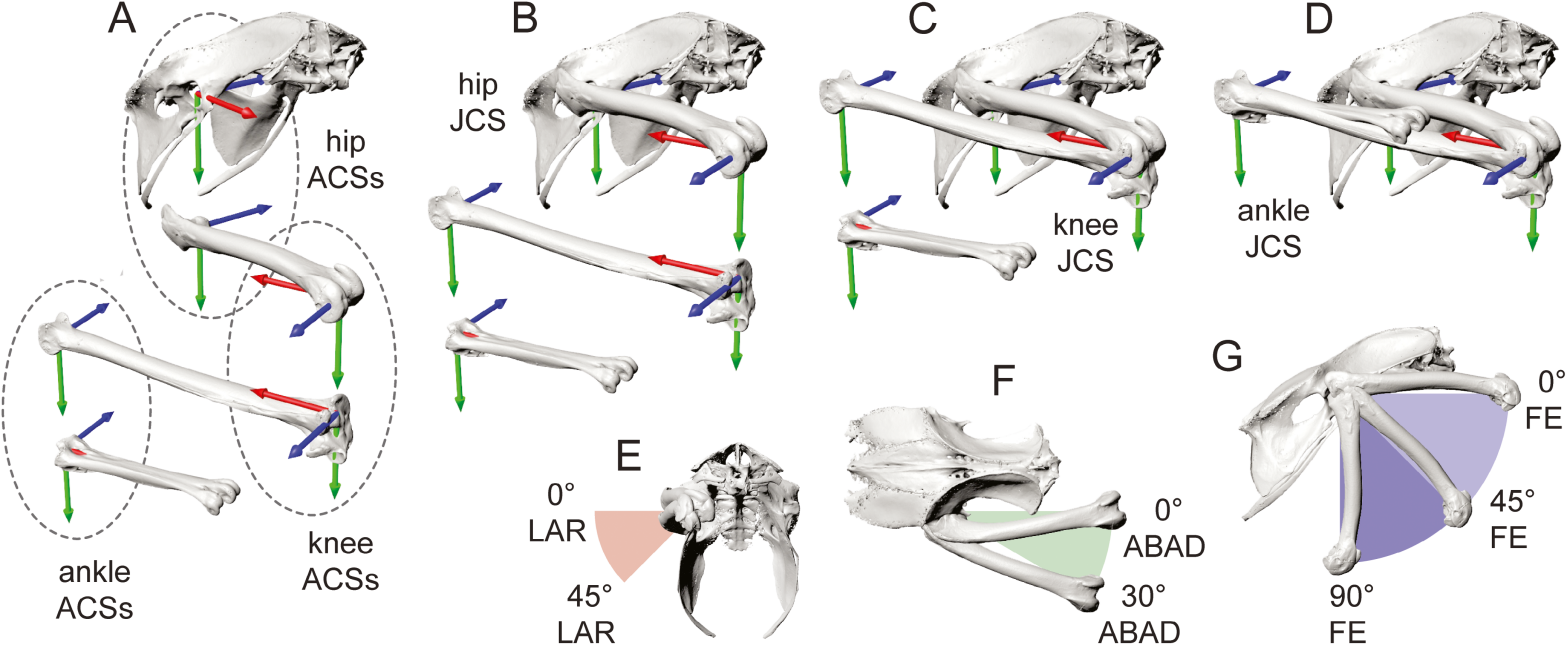
Combining pairs of anatomical coordinate systems (ACSs) to form joint coordinate systems (JCSs) and the reference pose, as demonstrated by a right guineafowl hindlimb in oblique views. (a) Each bone is oriented such that its ACSm is aligned with its mating ACSf on the more proximal segment, but translated vertically downward. (b) Completely registering the two hip ACSs puts the femur into its reference pose relative to the pelvis (zero hip translations and rotations). (c) Registering the two knee ACSs likewise adds the crus to the reference pose. Note that because the femoral condyles are skewed relative to the femoral long axis, the crus is not parallel to the femur in dorsal view. (d) Registering the two ankle ACSs completes the reference pose for the right hindlimb. Examples of long‐axis rotation (LAR) (e), abduction–adduction (ABAD) (f), and flexion–extension (FE) (g) rotations of the right hip

The reference pose represents the relationship among segments when JCSs are “zeroed out” in both translations and rotations. Registering the fixed and mobile ACSs at each joint puts the hindlimb into a configuration with the femur and foot pointing cranially and the crus pointing caudally relative to the pelvis (Figure 7a–d). The reference pose designates the initial position and orientation of segments from which translations and rotations are measured (Figure 7e–g). We chose to fully collapse the limb so that most in vivo poses would entail extension away from the reference pose and thus have positive FE values (Figure 7g). This coincides with quantification of FE in countless previous studies (e.g., Fischer, 1994; Fischer et al., 2002; Gatesy, 1991, 1999; Nyakatura et al., 2010) in which positive joint angles are measured cranial to the hip and ankle, but caudal to the knee.

**FIGURE 8:**
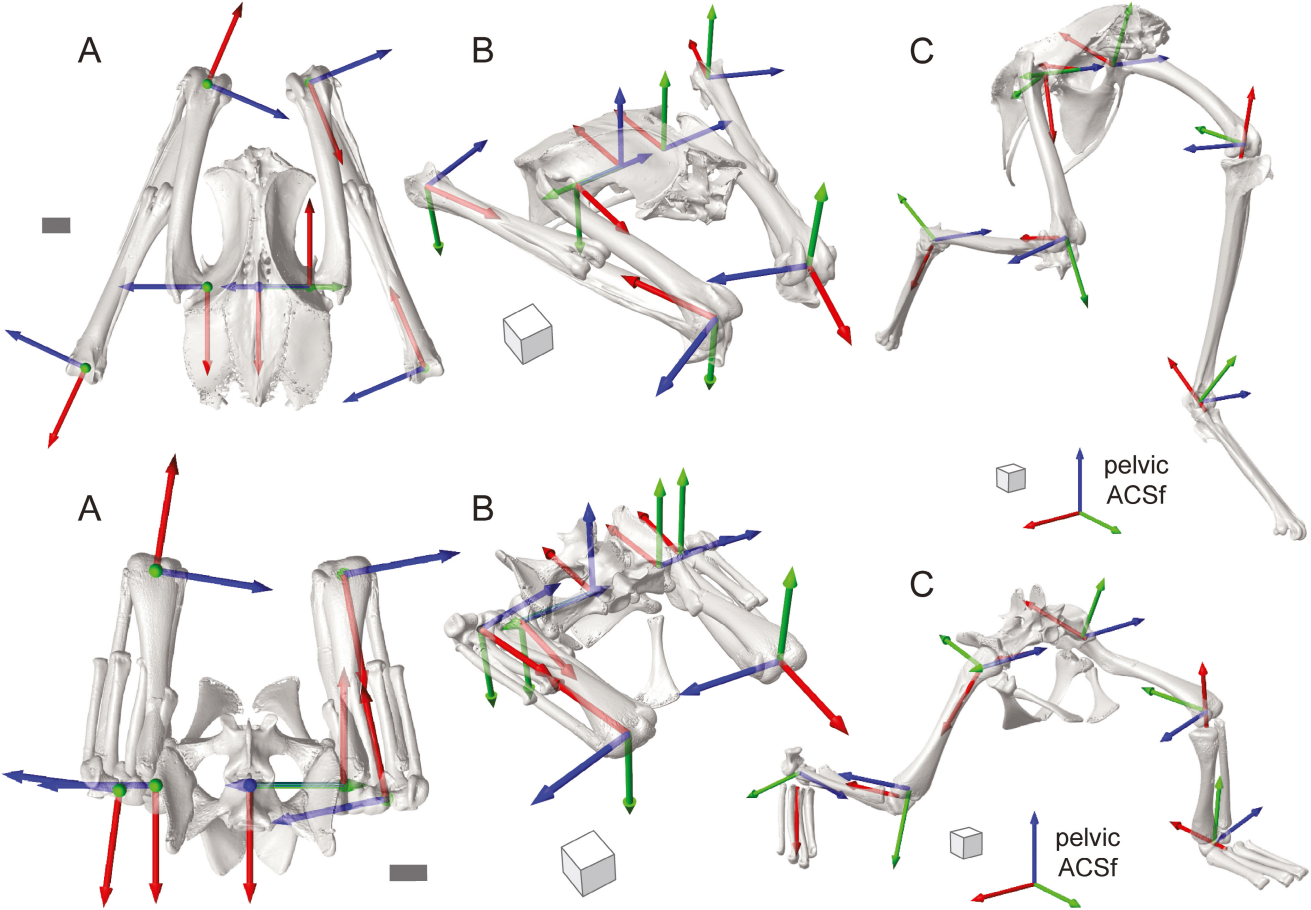
Joint coordinate system (JCS) axes in reference and locomotor poses, as demonstrated by guineafowl (top) and alligator (bottom) in dorsal (a) and oblique (b, c) views. In the reference poses (a, b), each anatomical coordinate system (ACS) pair is aligned to define zero translations and rotations. The limbs are collapsed into a tight Z‐configuration with unrealistic bone interpenetration. During locomotion (c), JCS translations both raise the pelvis above the ground‐based pelvicACSf and separate the articular surfaces at each joint. Scale bars and cube sides equal 1 cm

However, because our reference poses are created from anatomically derived ACSs that are influenced by joint morphology, they can appear to differ slightly among taxa (Figure 8a,b). For example, skewing of the femoral condyles relative to the femoral long axis (more obvious in guineafowl than alligator) causes the crus and foot to be angled away from the femur and pelvis (Figures 7d and 8a,b) in dorsal view. Such deviations represent a deliberate effort to integrate important aspects of articular morphology into the creation of ACSs, and why we make two per segment. This exemplifies what we mean by “joint‐inspired” in our standard’s fourth founding principle. In the case of the knee, we assert that kinematics are most comparable when the bicondylar nature of this joint is incorporated into its JCS. Thus, in our proposed standard the reference pose is not a predefined template into which we force each segment of the hindlimb skeleton. Rather, our seemingly dissimilar reference poses belie an underlying unanimity resulting from creating and aligning ACSs as consistently as possible.

Although JCS translations and rotations can be calculated directly from ACS pairs without first placing the skeleton into its reference pose, we consider doing so a good practice that offers several advantages. (Figure 8a,b). The reference pose serves as a simple visual check for ACS/JCS consistency. Within an individual, differences between right and left limb poses signal one or more asymmetries in the choice of surface patches, the fitting of geometric primitives, the calculation of axes by vector math, or the underlying skeletal morphology itself. Likewise, reference pose comparison within a species can help justify combining data from multiple individuals.

Our one major deviation from the human ISB standards for ACS/JCS creation is the absence of left–right symmetry. Following Kambic et al. (2014), we opted for asymmetrical JCSs so that equivalent anatomical motions are measured as rotations of equal magnitude and sign in both legs. For example, in the reference pose, the right hipJCS_Y points ventrally, whereas the left hipJCS_Y points dorsally (Figure 8a,b). Following the right‐hand rule, femoral abduction (moving away from the pelvic midline) is thus measured as a positive increase in ABAD rotation at both hips. If symmetrical (e.g., both hipJCS_Ys pointing ventrally) positive ABAD rotations would indicate abduction of the right hip, but adduction of the left. Femoral long axes are likewise represented asymmetrically. The right hipJCS_X points cranially and the left caudally, allowing external LAR to be positive in both. Note that both right and left hipJCS_Zs point to the left, thereby allowing hip extension to be measured as an increase in FE rotation, regardless of side. Ankle JCSs follow the hip JCS pattern, but kneeJCSs differ. Both kneeJCS_Z axes point to the right, so that extension is again measured as an increase in FE rotation. As at other joints, we set up kneeJCS_Xs to allow external LAR to be positive. However, in order for the knee ACSs to be right‐handed coordinate systems, the signs of ABAD must differ (abduction negative, adduction positive) from the hip and ankle.

When in the reference pose, each JCS can be shown as a single set of axes because fixed and mobile ACS pairs are fully registered (Figures 7d and 8a,b; Files S1 and S2). Representing a JCS graphically in expanded limbs is somewhat challenging, because different components are required for translations and rotations. Actual locomotor poses (reconstructed from biplanar X‐ray videos by XROMM from trials in Kambic et al., 2014; Tsai, Turner, et al., 2020) are shown in Figure 8c along with JCS axes about which joint rotations are calculated. Blue JCS_Z axes for measuring FE at the hips, knees, and ankles are fixed to their respective proximal segments (pelvis, femur, crus). Red JCS_X axes for measuring LAR at these joints travel with their respective distal segments (femur, crus, foot). Green JCS_Y axes for measuring ABAD are shown sharing an origin with JCS_Z axes, but this choice is arbitrary. The exact location of the JCS_Y axis does not affect calculation of ABAD angle. We chose not to represent the elements needed to calculate joint translations because these have already been described in detail. For the pelvicJCS pseudo‐joint, we show the ground‐based pelvicACSf (Figure 8c), which has not been figured previously.

## 4 DISCUSSION

**FIGURE 9:**
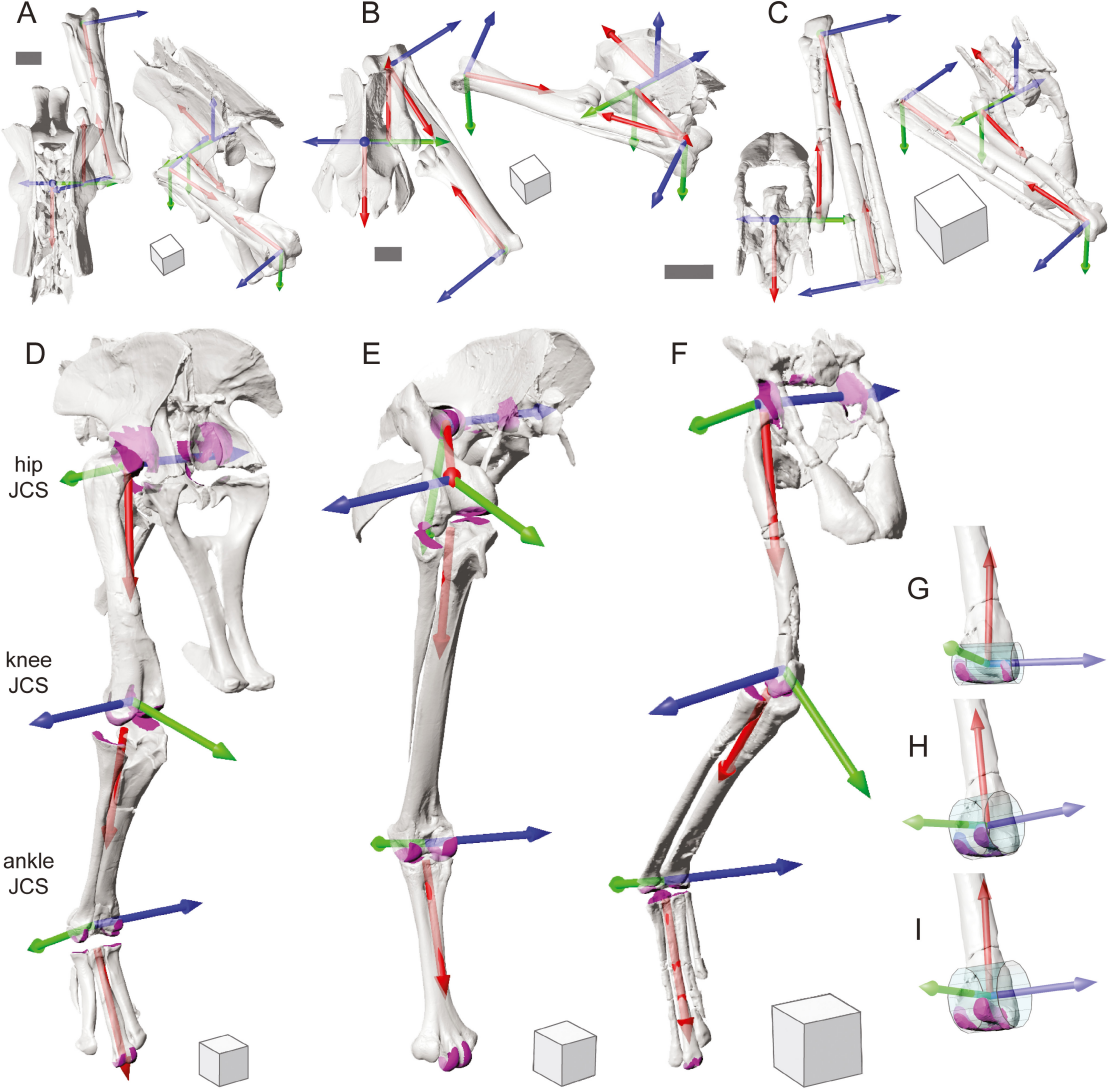
Joint coordinate system (JCS) axes applied to the right hindlimbs of three fossil taxa using the proposed standards. Reference poses for the extinct theropod, *Allosaurus* (a), the extinct bird, *Dinornis* (b), and the dinosauriform, *Marasuchus*/*Lagosuchus* (c) in dorsal and oblique views. (d–f) The same taxa posed as in Figure 1. Surface patches for creating coordinate systems for the pelvis, hip, knee, and ankle are shown in magenta. The pelvic JCSs have been omitted for clarity. (g–i) Three hypotheses of condyle homology on the distal right femur of *Marasuchus*/*Lagosuchus* (in caudolateral view). Alternative surface patch selections (magenta) affect primitive cylinder fitting (aqua) and thus anatomical coordinate system (ACS) placement, yielding different JCS results at both knee and hip joints. Alternative i is used in f. Scale bars equal 10 cm in a and b and 1 cm in c. Cube sides equal 10 cm in a, b, d, e and 1 cm in c and f

Here we present a standard for measuring the 3‐D poses of the pelvis and major hindlimb segments in the clade Archosauria, based on five founding principles. Application of our proposed approach to diverse archosaur species is critical to its utility for comparative analysis. Following the steps described for selecting patches, fitting primitives, calculating T‐shaped principal vectors, and creating ACSs in our representative archosaur species (Figures 3, 4, 5, 6), we derived JCSs for the three extinct taxa illustrated in Figure 1 (Figure 9; Method S2)—the dinosauriform *Marasuchus*/*Lagosuchus lilloensis* (Sereno & Arcucci, 1994), the non‐avian theropod *Allosaurus jimmadseni* (Loewen, 2009; Madsen, 1976) and the moa *Dinornis robustus* (Bishop, 2015; Bishop et al., 2019). Measurements of translations and rotations of these specific poses do not hold special significance per se. However, the implementation of our standard on such fossil‐derived models demonstrates the potential to generalize this method for archosaurs beyond the two taxa shown in Section 3.

Our main goals in proposing this standard are to improve communication among researchers, help others undertake 3‐D analyses of pose and motion, and foster comparative study of locomotor evolution. We hope that our explicitly stated founding principles and detailed description of steps for creating each ACS will allow others to follow if they so choose. At the same time, there is no single best or correct method for all applications. We remain open to alternatives and innovation as long as these different coordinate systems are sufficiently characterized to be reproducible. Toward these ends, our proposed standard may serve as a reference.

It is equally important to be specific about what the numbers derived from our JCSs represent. A joint’s pose or change in pose through time (kinematics) is expressed as three translations and three rotations of one coordinate system relative to another. In the case of the knee, the six degrees of freedom measure the displacement and orientation of the kneeACSm on the proximal crus with respect to the kneeACSf on the distal femur. We go to great lengths to place these ACSs as consistently as possible based on anatomical features of skeletal models, thereby rendering JCS outputs in some way comparable among individuals and species. Whether such data are equivalent, independent, or homologous in an evolutionary sense remains an open question, particularly given the geometric constraints imposed by limb proportions and hip height during the stance phase of terrestrial locomotion (Gatesy & Pollard, 2011). We cannot endorse the direct transfer of joint angles among species without consideration of such constraints. Likewise, the quantitative reconstruction of ancestral pose states based on JCS‐derived angles must be done extremely judiciously. Nevertheless, measurement of joint poses derived by a consistent standard is a crucial first step toward more derived parameterizations of intersegmental coordination (Gatesy & Pollard, 2011).

For the proposed standard described here, we openly acknowledge that unresolved and subjective elements inevitably remain. Several of these inherent weaknesses are addressed in the following paragraphs. However, based on our own experience during the development of this approach (e.g., Bishop, Cuff, et al., 2021; Bishop, Michel, et al., 2021; Demuth et al., 2020; Kambic, Roberts, et al., 2017; Kambic et al., 2014, 2015; Manafzadeh & Gatesy, 2021; Manafzadeh et al., 2021; Turner & Gatesy, 2021; Wiseman et al., 2021) and that of the much larger human biomechanics community, we have no doubt that a set of reasonable standards is better than no conventions at all. The complexities of 3‐D kinematics are daunting enough that understanding, intuition, and clear communication are hard‐won. Toward these aims, standards can help move the field forward.

A first caveat is the nature of surface patch selection. For example, variation in articular morphology can raise questions about homology across Archosauria. We encountered this issue with several joints, but the ambiguity among distal femoral condyles of *Marasuchus*/*Lagosuchus* is illustrative (Figure 9g–i). As is typical of early archosaurs, the lateral condyle of the femur in *Marasuchus*/*Lagosuchus* is substantially smaller than that part which is homologous to the tibiofibular crest of birds (Nesbitt, 2011; Parrish, 1986; Pintore et al., 2021). Given that the relative sizes of the two eminences on the lateral distal femur progressively changed along the avian stem lineage, which should be selected for *Marasuchus*/*Lagosuchus*? As shown in Figure 8, different surface patches yield different cylinder fittings, and thus different kneeACSf axes.

Joint coordinate systems derived from these three alternatives will yield quantitatively different knee poses for the same femur–crus relationship. Moreover, our segment‐based approach means that the three hipACSm axes will also diverge and produce different hip rotations. However, such uncertainty is not unique to our standard. In fact, we view our ability to visualize and quantify these differences as a strength, rather than a weakness, of our approach. Workers can explicitly show their choices and directly relate alternative hypotheses of surface homology to measurable deviations in pose. We do not advocate here which selection is “correct” (Figure 9c shows option I), but wish to highlight the ongoing challenges that variation can raise across a large clade.

Beyond questions of homology, surface selection will still involve some subjectivity. The fairly consistent morphology of humans enables the development of fully automated workflows (e.g., Miranda et al., 2010; Modenese & Renault, 2021; Renault et al., 2018), but disparity prevents such automation across Archosauria, where selection is a manual exercise. Yet a lack of repeatability need not all be due to user error. Our approach can reveal subtle individual variation (e.g., degree of femoral anteversion) that may impact intraspecific comparisons (e.g., Kambic, Roberts, et al., 2017). Likewise, studies of extinct taxa will likely encounter incomplete specimens and taphonomic degradation. Even if the articular surfaces are well preserved, deformation of the intervening diaphysis can result in skewed ACSs (Bishop, Cuff, et al., 2021). The effects of variable cartilage development (e.g., the femora of sauropods versus theropods; Tsai, Middleton, et al., 2020) may create problems for broad comparisons but also present an exciting opportunity to analyze the impact of soft tissues on how we characterize and communicate joint function (e.g., Tsai, Turner, et al., 2020). The types of impacts and their consequences for downstream quantitative interpretation will likely vary on a case‐by‐case basis. Appropriate sensitivity testing can be done to assess whether pose quantification is reliable enough to address a study’s core questions.

We recognize a second caveat on a more technical level. The use of Euler/Tait–Bryan angles for pose quantification is fundamentally constrained by trigonometric nonlinearity. Unlike translations, the effect of a unit of angular displacement on the orientation of a rigid body can depend on the body’s current orientation. This leads to the well‐known problem of mathematical singularity when the second rotation (*Y* in our approach; ABAD or pelvic pitch) reaches ±90°; at this point the *X* and *Z* axes become colinear and a degree of freedom is lost (“gimbal lock”), rendering it impossible to uniquely describe the body’s attitude. The recent implementation of cosine‐corrected Euler space (Manafzadeh & Gatesy, 2020) resolves Euler angle nonlinearities, insofar as they relate to measuring joint mobility as a volume in 3‐D pose space, but the singularity problem is unavoidable.

Ideally, ACSs and JCSs should be defined such that, under known or expected in vivo conditions, poses that approach the singularity are not encountered. This is not always possible, however, particularly at highly mobile joints. The convention outlined above is optimized for adducted (erect) postures, but as the limb becomes more abducted (sprawled) hip ABAD nears 90° and our JCS approaches a singularity. The same problem arises if the pelvis pitches too far up or down. No one system of Euler/Tait–Bryan angles works well for all postures, which presents a unique challenge for studies investigating functional evolution across a broad postural continuum.

A third set of caveats stem from the spatial scale at which we construct ACSs and JCSs. Our “T method” draws upon information from both proximal and distal ends of a limb segment in deriving ACSs. Therefore, not only are the proximal and distal ACSs of a given segment dependent on one another, two whole segments are needed to derive a single JCS at their shared joint. The requirement for whole bones places a constraint on studies of extinct species where specimen completeness may be a problem. For example, quantifying knee poses (via a knee JCS) requires a whole femur and whole crus, regardless of whether the hip or ankle is also a joint of interest. We welcome researchers developing methods for generating ACSs with less or even no reliance on the segment’s long axis (e.g., Carney, 2016) that can be applied to more fragmentary material.

Lastly, by taking a segment‐based perspective, our approach currently only characterizes the translational and rotational offsets *between* major limb segments. Relative motion of individual bones *within* a segment, such as between the patella and femur (Allen et al., 2017), fibula and tibia (Fuss, 1996), or adjacent metatarsals (Turner & Gatesy, 2021) is known to occur in extant archosaurs. We do not consider such movements unimportant or unworthy of investigation. Rather, the approach outlined here serves as a practical framework for expansion, exploration, and refinement. Such flexibility also means that it is important to be maximally transparent about how the approach is applied in future studies.

Despite the caveats noted above, the general philosophy outlined here sets up the foundation needed to start looking at key questions regarding limb pose and motion across archosaurs, and potentially beyond this clade. Such broader applicability might be enabled by the gross similarity of joint articular surfaces across many tetrapod lineages (e.g., Romer, 1956), but this remains to be explored. There are far more potential applications even within Archosauria—most notably evolutionary explorations of in vivo*/*ex vivo joint kinematics and mobility (e.g., Manafzadeh et al., 2021) and mechanics. Adopting the common “language” proposed here should grant not only kinematics but also kinetics (e.g., joint moments) more “apples‐to‐apples” compatibility within (i.e., ontogenetically/intraspecifically) and across taxa. This compatibility is also more clearly communicated by our methodology.

Our standards hold potential for illuminating data from other approaches as well. For example, geometric morphometric analyses of joint shape could be combined with JCS‐derived joint pose spaces (akin to Manafzadeh et al., 2021) to quantitatively relate articular geometry with higher level functional inferences in a phylogenetic context (e.g., Harcourt‐Smith et al., 2008). Landmark‐based analyses (e.g., Lawing & Polly, 2010; Mitteroecker & Gunz, 2009) might then pinpoint which specific features altered (or conserved/constrained) ACS/JCS parameters in archosaurs (Pintore et al., 2021). Such ambitious integrations could yield major advances in our understanding of fundamental form‐function relationships across development, evolution, and phenotypic plasticity (e.g., Simons et al., 2019).

For example, one major transformation that has long drawn the attention of paleontologists is the evolution of tarsal morphology and articulations in archosaurs (e.g., Brinkman, 1980; Cruickshank, 1979; Cruickshank & Benton, 1985; Demuth et al., 2020; Parrish, 1987, 1993; Sereno & Arcucci, 1990). Our approach will enable an explicit, more objective means of placing these morphofunctional transformations into a quantitative context, perhaps better explaining how “crocodile‐normal”, “crocodile‐reversed”, and “advanced mesotarsal” ankle forms do or do not differ from each other, and how these variants evolved. Additionally, poses in physical or digital skeletal mounts could be clearly compared and assessed, even in the design phase, aiding museum exhibition as well as research via quantitative assessment.

Finally, the 3‐D nature of our approach is extremely amenable for applying it to questions about 3‐D control of joints by the neuromuscular system. If standard‐derived JCS axes serve as the basis for rigging virtual joints, studies using forward‐kinematic or inverse‐kinematic “digital marionettes” can predictably quantify animated stride or wingbeat cycles. Even more complex dynamic musculoskeletal simulations could also adopt this approach, much as human studies generally have adopted the “ISB standard” pioneered by Grood and Suntay (1983) for a diversity of analogous applications. We see a bright future for studies of archosaurian motion illuminated by this proposed approach and the augmentations that it inspires.

## AUTHOR CONTRIBUTIONS

S.M.G. conceived the study. S.M.G., A.R.M., P.J.B., M.L.T., and R.E.K. contributed to content concept/design. A.R.M. and P.J.B. acquired data. S.M.G., A.R.M., P.J.B., M.L.T., A.R.C., and J.R.H. carried out data analysis/interpretation. S.M.G., A.R.M., P.J.B., M.L.T., and J.R.H. drafted the manuscript. All authors critically revised and approved the manuscript prior to submission.

## Supporting information


Supplement Material

## Data Availability

Files S1 and S2 are available for download from the XMAPortal at: https://xmaportal.org/webportal/larequest.php?request=CollectionViewAllFiles&StudyID=71&instit=BROWN&collectionID=19. Copies of the Allosaurus and Dinornis bone models have been accessioned with the Paleontology Collections of the Museum of the Rockies and the Natural History Collections of the Canterbury Museum, respectively; they can be freely accessed by contacting the Curator or Collections Manager (john.scannella@montana.edu, info@canterburymuseum.com). Custom Maya Embedded Language scripts for importing fit geometric primitives from Geomagic Studio/Wrap are available at: https://bitbucket.org/xromm/xromm_other_mel_scripts/src/main/
