# Supplementary material for: A proposed standard for quantifying 3‐D hindlimb joint poses in living and extinct archosaurs
Source: J Anat. 2022 Feb 3;241(1):101–18. doi: 10.1111/joa.13635 (PMC9178381; doi:10.1111/joa.13635)
Supplement: Supplementary file 1 — Supplement Material [file JOA-241-101-s001.docx]

**SUPPLEMENTARY MATERIAL**

**Supplementary File S1.** Autodesk Maya scene containing resulting Helmeted Guineafowl surface patch selections, geometric primitive fits, and coordinate systems.

**Supplementary File S2.** Autodesk Maya scene containing resulting American alligator surface patch selections, geometric primitive fits, and coordinate systems.

Supplementary Files S1-2 are available for download from the XMAPortal at: https://xmaportal.org/webportal/larequest.php?request=CollectionViewAllFiles&StudyID=71&instit=BROWN&collectionID=19

**Supplementary Method S1. Example of Autodesk Maya code for anatomical coordinate system creation and placement**

Although several combinations of software can be used to select surface patches, fit geometric primitives, and create and place coordinate systems on skeletal models, here we offer an example of the Maya Embedded Language (MEL) code that we used in Autodesk Maya to place both anatomical coordinate systems (rhipM and rkneeF) on a right femur model. Using this procedure requires that fit geometric primitives have been imported into Maya with the proper transformations obtained from primitive fitting software. MEL scripts to import fit spheres, cylinders, and planes from Geomagic Studio/Wrap have been made available as part of this publication at: <https://bitbucket.org/xromm/xromm_other_mel_scripts/src/main/>

In the example code below, the distal femoral condyle fit cylinder has been imported as an object named fitCylinder, and the proximal femoral head fit sphere has been imported as an object named fitSphere (both indicated in red text in the code below). Note that proper functioning of this code relies on a script contained within the XROMM_mayaTools package, available for download at xromm.org.

{

//************extract information from femoral geometric primitives************

//extract axis vector and centroid from distal femoral condyle cylinder fit, where cylinder is an object named fitCylinder

float $cylinderMatrix[16] = `xform -q -ws -m fitCylinder`;

vector $cylinderAxis = unit(<< $cylinderMatrix[8],$cylinderMatrix[9],$cylinderMatrix[10] >>);

vector $cylinderCentroid = << $cylinderMatrix[12],$cylinderMatrix[13],$cylinderMatrix[14] >>;

//cylinder axis vector must be facing to the right for proper signs, include this line ONLY if axis vector is facing left to flip it to face right

$cylinderAxis = -$cylinderAxis;

//extract centroid from femoral head sphere fit, where sphere is an object named fitSphere

vector $sphereCentroid = `xform -q -ws -t fitSphere`;

//set a length and radius for the ACSs to be constructed -- modify these values as desired

float $ACSlength = 1;

float $ACSradius = 0.1;

//************create rhipM ACS************

//create the X vector pointing distally by subtracting the sphere centroid from the cylinder centroid

vector $X = unit($cylinderCentroid - $sphereCentroid);

//create the Y vector pointing down by crossing $X and $cylinderAxis

vector $Y = unit(cross($X, $cylinderAxis));

//create the Z vector pointing left by crossing $X and $Y

vector $Z = unit(cross($X, $Y));

//create a set of axes named rhipM -- this function requires the XROMM_mayaTools package from xromm.org

createGiantHemilocatorHR ("rhipM", $ACSlength, $ACSradius);

//transform ACS to the correct position ($sphereCentroid) and orientation (based on $X, $Y, $Z) by building a matrix

xform -ws -m ($X.x) ($X.y) ($X.z) (0)

($Y.x) ($Y.y) ($Y.z) (0) ($Z.x) ($Z.y) ($Z.z) (0)

($sphereCentroid.x) ($sphereCentroid.y) ($sphereCentroid.z) 1 rhipM;

//************create rkneeF ACS************ //create the $Z vector pointing right from $cylinderAxis

vector $Z = $cylinderAxis;

//create a temporary X vector pointing distally by subtracting the sphere centroid from the cylinder centroid

vector $tempX = unit($cylinderCentroid - $sphereCentroid);

//create the Y vector pointing down by crossing $tempX and $Z

vector $Y = unit(cross($tempX, $Z));

//create the X vector pointing proximally by crossing $Y and $Z

vector $X = unit(cross($Y, $Z));

//create a set of axes named rkneeF -- this function requires the XROMM_mayaTools package from xromm.org

createGiantHemilocatorHR ("rkneeF", $ACSlength, $ACSradius);

//transform ACS to the correct position ($cylinderCentroid) and orientation (based on $X, $Y, $Z) by building a matrix

xform -ws -m ($X.x) ($X.y) ($X.z) (0)

($Y.x) ($Y.y) ($Y.z) (0) ($Z.x) ($Z.y) ($Z.z) (0)

($cylinderCentroid.x) ($cylinderCentroid.y) ($cylinderCentroid.z) 1 rkneeF;

}

**Supplementary Method S2. Description of methods used to generate skeletal geometries for extinct taxa**

*Marasuchus*/*Lagosuchus lilloensis*

The model was constructed as a composite of two roughly similarly sized, presumed adult individuals PVL 3870 and PVL 3871 (Paleontología de Vertebrados, Instituto Miguel Lillo, Tucumán, Argentina), with elements from PVL 3871 scaled to the size of PVL 3870, which preserved the more complete pelvis and sacrum. Limb bones from both sides were used, based on completeness and preservation. Geometry was obtained via micro-CT scanning (SkyScan 1173 [Bruker, Massachusetts], ~80 kV, ~65 μA; ~1120x1120 resolution; 50-70 μm pixel size) and segmentation in Mimics software (Materialise, Inc., Leuven, Belgium).

The distal half of the pubis was taphonomically bent slightly medially, which prevented anatomically accurate articulation of the pubis and ischium with their (mirrored) counterparts. Thus, the distal pubis was retrodeformed using the “cage edit” tool in Rhinoceros 4.0 (McNeel, Seattle, USA), a form of host-mesh warping; only the minimal amount of lateral unbending was used to construct a properly articulated, whole and symmetrical pelvis.

Due to the asymmetry in shape and location of the distal tarsals, the proximal tarsometatarsus did not form a level articular surface, precluding easy selection of patch areas for primitive fitting. Therefore, the whole proximal aspect of this bone complex was used to fit a plane, which insofar as deriving a centroid did not appear to importantly affect the end result.

*Allosaurus jimmadseni*

The model was constructed from a single “subadult” specimen, MOR 693 (Museum of the Rockies, Bozeman, USA). The model is a composite of elements from both sides of the body, which were selected based on completeness/preservation quality or their accessibility for digitization: left ilium, right ischium, left and right pubes, sacral vertebrae 1–5 (+ caudosacral 1 still in articulation); right femur, tibia, fibula, astragalus and calcaneum; left distal tarsal III, right distal tarsal IV; right metatarsal I, left metatarsals II–V; right digit I, left digits II–IV. Limb bone geometry was captured using computed tomographic scanning (full details previously outlined by Bishop et al., 2018) whereas the geometry of pelvic and sacral elements was captured using digital photogrammetry (Reality Capture; Capturing Reality s.r.o., Slovakia).

The paired pubes were articulated as in life, but had suffered a modest amount of plastic taphonomic distortion. It was evident that such distortion could be approximated as a simple shearing of the bones in the coronal plane. Therefore, the major distortion was corrected by “unshearing” of the bones in the coronal plane, with the shear axis parallel to the mediolateral direction, was undertaken using the “shear” tool in the software Rhinoceros. Finally, the proximal pubes had also evidently collapsed in toward the midline; the articulating facets of the iliac peduncles faced somewhat inwards, rather than directly dorsally. Thus, the proximal part of both pubes was bent away from the midline to produce a roughly symmetrical pubic façade, achieved using the cage edit tool in Rhinoceros. The pelvis and sacrum was then able to be effectively articulated, using the apposing facets of the peduncles and sacral rib scarring as a guide, and by creating mirror images of the singular ilium and ischium. With the exception of the pubes, no other limb element used to construct the model exhibited any evidence for taphonomic deformation that would affect the results of the present study.

Distal tarsals III and IV were re-articulated with the proximal metatarsals following the reconstruction illustrated by Madsen (1976) for *Allosaurus fragilis*, but they were not used in the computation of the proximal metatarsus plane.

The selection of surfaces of the sacral centra for cylinder fitting was slightly different from what has been used elsewhere. This was due to the presence of a metal armature supporting the specimen, but a cylinder still fit acceptably well.

*Dinornis robustus*

The model was constructed primarily from a single adult specimen, CM Av8422 (Canterbury Museum, Christchurch, New Zealand). All bones except the fibula came from this individual; the fibula of a similarly sized second individual from the same locality, CM Av8488 (left side), was used. The limb bones from CM Av8422 were sourced from both left and right sides: left femur, right tibiotarsus, left tarsometatarsus, right metatarsal I, right phalanges (except IV-5, which was substituted with the left). The geometries of the pelvis, femur, tibiotarsus, fibula and tarsometatarsus were acquired using CT scanning as part of previous studies (Bishop, 2015; Bishop et al., 2019); the phalanges and metatarsal I were acquired via digital photogrammetry (Agisoft Photoscan, Agisoft LLC, St Petersburg, Russia). All bones are pristinely preserved, and did not exhibit any evidence of taphonomic deformation.

In deriving the pelvic ACS, sacrals 10 and 16, but not those in between, were used for patch selection and cylinder fitting. This was necessitated by the marked difference in sacral anatomy between *Dinornis* and *Numida*, in particular the massive and closely packed sacral ribs that renders the ventral surface of this part of the sacrum essentially flat.

Copies of the *Allosaurus* and *Dinornis* bone models have been accessioned with the Paleontology Collections of the Museum of the Rockies and the Natural History Collections of the Canterbury Museum, respectively; they can be freely accessed by contacting the Curator or Collections Manager (john.scannella@montana.edu, info@canterburymuseum.com).

*Supplementary References*

**Bishop**, P.J. (2015) A critical re-evaluation of the hindlimb myology of moa (Aves: Dinornithiformes). *Memoirs of the Queensland Museum*, 59, 187–246.

**Bishop**, P.J., **Hocknull**, S.A., **Clemente**, C.J., et al. (2018) Cancellous bone architecture and theropod dinosaur locomotor biomechanics. Part I – the potential utility of cancellous bone, with a comparative study of cancellous bone architecture in the hindlimb bones of theropods. *PeerJ*, 6, e5778.

**Bishop**, P.J., **Scofield**, R.P. & **Hocknull**, S.A. (2019) The architecture of cancellous bone in the hindlimb of moa (Aves: Dinornithiformes), with implications for stance and gait. *Alcheringa*, 43, 612–628.

**Madsen**, J.H., Jr. (1976). *Allosaurus fragilis*: A revised osteology. *Bulletin of the Utah Geological Survey*, 109, 1–163.
